# Exploring sleep health and circadian rhythm disruption in Sjögren’s disease: an accelerometric and self-reported cross-sectional study

**DOI:** 10.1093/rheumatology/keag134

**Published:** 2026-03-21

**Authors:** Caterina Di Pede, Alessandro Colitta, Simone Bruno, Paolo Frumento, Michelangelo Maestri Tassoni, Giovanni Fulvio, Gaetano La Rocca, Enrica Bonanni, Marta Mosca, Rosaria Talarico, Chiara Baldini, Ugo Faraguna

**Affiliations:** Department of Translational Research and of New Surgical and Medical Technologies, University of Pisa, Pisa, Italy; Department of Clinical and Experimental Medicine, Neurology Unit, University of Pisa, Pisa, Italy; Department of Psychiatry, University of Wisconsin-Madison, Madison, WI, United States; Department of Political Sciences, University of Pisa, Pisa, Italy; Department of Clinical and Experimental Medicine, Neurology Unit, University of Pisa, Pisa, Italy; Department of Clinical and Experimental Medicine, Rheumatology Unit, University of Pisa, Pisa, Italy; Department of Clinical and Experimental Medicine, Rheumatology Unit, University of Pisa, Pisa, Italy; Department of Clinical and Experimental Medicine, Neurology Unit, University of Pisa, Pisa, Italy; Department of Clinical and Experimental Medicine, Rheumatology Unit, University of Pisa, Pisa, Italy; Department of Clinical and Experimental Medicine, Rheumatology Unit, University of Pisa, Pisa, Italy; Department of Clinical and Experimental Medicine, Rheumatology Unit, University of Pisa, Pisa, Italy; Department of Translational Research and of New Surgical and Medical Technologies, University of Pisa, Pisa, Italy; Department of Developmental Neuroscience, Istituto di Ricovero e Cura a Carattere Scientifico (IRCCS) Foundation Stella Maris, Pisa, Italy

**Keywords:** Sjögren’s syndrome, quality of life, depression, outcome measures, nervous, wider determinants of health, behaviour

## Abstract

**Objectives:**

In a cross-sectional study, we aimed at characterizing possible impairments in sleep health and rest–activity parameters between patients with Sjögren’s Disease (SjD) and healthy controls (HCs). Furthermore, we explored possible predictors of such disturbances in the SjD group.

**Methods:**

Participants’ sleep and rest-activity rhythms were assessed via 7-day continuous accelerometry, the Pittsburgh Sleep Quality Index, the Epworth Sleepiness Scale, and the reduced Morningness-Eveningness Questionnaire, allowing the creation of a multidimensional sleep health index. Parametric tests explored between-group differences in sleep and rest-activity parameters, while functional linear modelling characterized between-group, time-related differences in accelerometric activity. Within the SjD cohort, regression analysis was employed to explore disease activity, patient-reported disease burden, and the Hospital Anxiety and Depression Scale (HADS) as possible predictors of sleep and rest-activity parameters.

**Results:**

Forty-six SjD patients and forty age-, sex- and BMI-matched HCs were included. Compared with HCs, SjD patients reported lower sleep health (*P* = 0.005) and delayed mid-sleep point (*P* = 0.009) and acrophase (*P* = 0.033). Functional linear modelling confirmed the objective shift towards eveningness in SjD patients. In SjD patients, patient-reported disease burden predicted sleep quality (β = 0.41; *P* = 0.044) and sleepiness (β = 0.83; *P* = 0.010), while disease activity predicted daily steps (β=−526; *P* = 0.013), total sleep time (β = 0.15; *P* = 0.0496) and sleep regularity (β=−1.3; *P* = 0.030). Finally, HADS predicted daily steps (β = −238; *P* = 0.041), total sleep time (β = −0.08; *P* = 0.035) and sleep efficiency (β = −0.65; *P* = 0.012).

**Conclusions:**

SjD is associated with impaired sleep health and rest-activity rhythms. In SjD patients, such alterations differentially associate with disease activity and patient-reported outcomes, supporting a multifactorial model of sleep and circadian rhythms disruption.

Rheumatology key messages

- Sjögren’s disease patients show impaired sleep health and rest-activity parameters compared to healthy controls.
- SjD patients show delayed circadian phase compared to healthy controls, as measured by accelerometry.
- SjD patients’ sleep and rest-activity parameters differentially associate with disease activity and patient-reported outcomes.

## Introduction

Sjögren’s Disease (SjD) is a systemic autoimmune disorder primarily affecting the exocrine glands, leading to xerostomia and xerophthalmia (sicca symptoms) [1]. Beyond glandular involvement, SjD includes systemic features such as fatigue, musculoskeletal pain, and autonomic dysfunction [1]. Sleep disturbances and daytime sleepiness are highly prevalent in SjD, with rates ranging from 38% to 83% [2, 3]. While previous research has identified patient-reported symptom severity, disease activity, pain, anxiety, and depression as key factors in SjD sleep disturbances [2, 4–7], the association between higher disease activity and altered sleep remains inconsistent [2, 5, 8, 9]. This suggests that non-immunologic factors may play a pivotal role in SjD patients’ sleep disturbances.

To date, the evaluation of sleep in SjD has largely relied on subjective tools, such as the Pittsburgh Sleep Quality Index (PSQI) and the Epworth Sleepiness Scale (ESS) [2, 4, 9], with limited research employing objective sleep assessment methods. In these studies, polysomnography (PSG) showed several altered sleep macroarchitecture parameters in SjD patients, including prolonged sleep latency [10] and increased nocturnal awakenings [6]. Of note, only 3 studies employed accelerometry, a non-invasive sleep-wake assessment method which can provide quantitative sleep and circadian parameters in ecological settings, based on relatively long-term activity monitoring [11–13].

Both subjective and objective sleep parameters have been recently included in the estimation of a multidimensional sleep health index [14–16]. Sleep health, as defined by *Buysse* (2014), is a multidimensional construct encompassing 6 dimensions of sleep: regularity, satisfaction, alertness, timing, efficiency, and duration. When performing longitudinal population studies, the alteration of any one of these dimensions emerged as an independent predictor of adverse health outcomes, including higher mortality [17–19], cardiometabolic morbidity [15, 16], and increased systemic inflammation markers [20]. Irrespective of such potential impact of patients’ systemic inflammation and health, sleep health has never been investigated in SjD.

Growing evidence supports the role of rest-activity circadian rhythms in modulating immune function being associated with higher systemic inflammatory markers [21]. Because of this, circadian rhythms have been recently explored in various autoimmune disorders [22–24]. However, no studies to date have examined rest-activity circadian patterns in patients with SjD, nor their potential associations with disease activity or patient-reported symptoms.

Within this context, we designed a cross-sectional study aiming at: (i) characterizing for the first time sleep health and circadian parameters in a sample of SjD patients using longitudinal, 7-day, continuous accelerometric monitoring and questionnaires; (ii) comparing sleep and circadian parameters between SjD patients and age-, sex-, BMI-matched healthy controls (HCs); (iii) investigating the relative contribution of patient-reported SjD symptoms, disease activity and mood and anxiety symptoms to sleep and circadian rhythm disruption in SjD patients.

## Materials and methods

### Eligibility criteria and ethics statement

Adult participants fulfilling the following inclusion criteria were recruited: providing the signed informed consent; being fluent in Italian; not being a shift worker. SjD patients additionally met the ACR/EULAR Classification Criteria for Sjögren’s Syndrome [25]. Individuals diagnosed with sleep disorders or rheumatologic, neurologic and psychiatric diseases were not included among healthy controls (HCs).

The study was approved by the Bioethical Committee of the University of Pisa (19233_MOSCA) and was conducted in compliance with the principles of the Declaration of Helsinki.

### Study design and procedure

A cross-sectional study was conducted. SjD patients were consecutively recruited during their visits at the Rheumatology Unit of the Pisa University Hospital between December 2024 and May 2025, when they underwent a comprehensive clinical evaluation investigating eligibility criteria, disease activity [26, 27], possible presence of fibromyalgia [28], and pharmacological history. Age-, BMI-, and sex-matched HCs were consecutively recruited after a clinical interview investigating their eligibility criteria within the same timeframe. Upon reaching the minimum sample size required per group (*n* = 39, see Data Analysis section), we prioritized strict matching for age, BMI, and sex over a perfect 1:1 patient-control ratio, aiming at ensuring the validity of between-group univariate comparisons.

Included participants were subsequently given both an accelerometer, to be continuously worn for a week, and a digital survey combining self-report questionnaires and basic questions on sociodemographic data. Self-report questionnaires explored participants’ sleep quality, sleepiness, and chronotype. SjD patients’ survey included also self-report questionnaires investigating severity of anxiety, depression, fatigue, and SjD-related symptoms. After the visits, medical records were systematically examined to gather SjD patients’ clinical data.

## Measures

### Sociodemographic and clinical data

The following sociodemographic data were collected: age, body mass index (BMI), sex, ethnicity, and shift working (yes/no). SjD disease activity was evaluated using the EULAR primary Sjögren’s syndrome Activity Index (ESSDAI). The ESSDAI evaluates SjD disease activity through 12 weighted items [26, 27]. The cut-off score for active disease is ESSDAI ≥ 5 [26]. Fibromyalgia was diagnosed based on the Analgesic, Anesthetic, and Addiction Clinical Trial Translations Innovations Opportunities and Networks–American Pain Taxonomy Diagnostic Criteria [28]. Furthermore, the following clinical information was gathered: disease duration (years), immunosuppressive and immunomodulatory treatment (yes/no).

### Self-report questionnaires

The EULAR primary Sjögren’s syndrome patient reported index (ESSPRI) was employed to investigate reported severity of SjD-related symptoms over the past 2 weeks [26, 27]. An unsatisfactory symptom state was defined as ESSPRI ≥ 5 [27]. The Hospital Anxiety and Depression Scale (HADS) is a self-administered questionnaire assessing the severity of anxiety and depression symptoms [29]. The Functional Assessment of Chronic Illness Therapy-Fatigue (FACIT) is a 13-item patient-reported outcome measure to assess the severity of fatigue [30]. Higher FACIT scores indicate lower fatigue [30].

The PSQI is a self-administered 19-item questionnaire measuring sleep quality over a one-month time interval [31]. A global score >5 is an index of poor sleep quality, defining an individual as a poor sleeper [31]. Self-reported bedtime and wake-up time were extracted from the PSQI, as assessed by Items 1 and 3. Daytime sleepiness was measured through the Epworth Sleepiness Scale (ESS) [32]. A global ESS score higher than 10 indicating excessive daytime sleepiness [32]. The reduced Morningness-Eveningness Questionnaire (rMEQ) is a self-administered questionnaire to estimate the chronotype of an individual [33]. The scale classifies scores as: evening type (<11), neither type [11–18], and morning type (>18). A thorough description of the administered questionnaires is provided in Supplementary Data S1.

### Accelerometry

All subjects continuously wore a Fitbit Inspire 2^®^ (FI2) for seven days on their non-dominant wrist, which enabled the collection of complete accelerometric time series. The FI2 smartband is a commercial device capable of tracking wrist activity through a micro-electro-mechanical systems triaxial motion sensor. Normalized, activity data were obtained through a dedicated Application Programming Interface (API) and digitally stored as normalized Activity Units with a 1-min resolution. Activity data were subsequently analysed through the *Dormi* algorithm by Sleepacta s.r.l., a validated and certified artificial neural network-based script [34] registered as a medical, risk class I device within the European Database on Medical Devices (EUDAMED) (UDI-DI: PP13374SLEEP78/IFA) [35]. The following quantitative accelerometric sleep parameters were estimated through the *Dormi* algorithm [14, 22–24, 36]:

- Total Sleep Time (TST): the total duration of sleep (hours). It is defined as the sum of all asleep time within a sleep period;
- Waking After Sleep Onset (WASO): the time spent awake within a sleep period (minutes);
- Sleep Efficiency (SE): the time spent asleep within a sleep period, expressed as a percentage of the sleep period (%). WASO and SE were considered as measures of sleep fragmentation [36];
- Sleep Regularity Index (SRI): the likelihood of the same sleep-wake state occurring in epochs that are 24 hours apart (%). SRI measures the similarity of sleep-wake patterns between consecutive days [36];
- Mid-sleep point: the middle of the sleep period between the sleep onset and final awakening, calculated by adding to the average sleep onset half of the average total sleep time (average sleep onset + average TST/2) [36].
- Sleep onset: the first episode of consolidated sleep, as defined by 10 consecutive 1-minute epochs of immobility [36]
- Sleep offset: the final epoch of the last episode of consolidated sleep in a sleep period [36]

To estimate parametric rest–activity circadian rhythm parameters, i.e., Midline Estimating Statistic of Rhythm (MESOR), amplitude, and acrophase [22, 23, 37], we applied the cosinor method by fitting a sine wave to the complete, normalized Activity Units time-series through the R package ‘Cosinor’ [38]. Non-parametric measurements of rest–activity rhythms, i.e., interdaily stability, intradaily variability, and relative amplitude [22, 23, 39], were computed using the R package Non-Parametric Measures of Actigraphy Data ‘nparACT’ [40]. When computing non-parametric measures, the analysis of time series was restricted to complete 24-h cycles anchored to the start time of the recording [40]. Variables are defined as follows:

- MESOR: a rhythm-adjusted mean. It represents the mean of the activity level as modelled by the sine wave;
- Amplitude: half of the peak-to-nadir difference, a measure of the extent of predictable variation within a cycle. More robust rhythms have a higher amplitude;
- Acrophase: timing of peak activity or the point in the cycle with highest activity;
- Interdaily stability (IS): estimates the variability in rest–activity patterns across all days. It is a measure of rest–activity rhythms regularity. It is expressed as values ranging from 0 to 1. Higher values indicate greater stability between days;
- Intradaily variability (IV): quantifies the fragmentation and magnitude of rest–activity transitions within each day. It is usually expressed as values ranging from 0 to approximately 2. Higher values indicate frequent transitions between rest and activity (i.e. frequent naps, increased night-time awakenings);
- Relative amplitude: measures the robustness of the 24 h rest–activity rhythm by calculating the normalized mean difference in activity between the most active 10 h and the least active 5 h, ranging from 0 to 1. Higher values indicate lower activity during the night and high activity during the day, i.e. increased robustness of rest–activity rhythm.

### The sleep health index

A sleep health composite index was computed based on the RU-SATED framework, a conceptual model defining sleep health as “a multidimensional pattern of sleep-wakefulness […] that promotes physical and mental well-being” [18]. The author identifies 6 dimensions of sleep, based on their association with negative health outcomes: regularity, satisfaction, alertness, timing, efficiency, and duration [18].

Multidimensional sleep health was previously validated as a longitudinal predictor of all-cause mortality [19] and was demonstrated to be associated with cardiometabolic morbidity [15, 16] and to mediate the association between evening chronotype and smoking behaviour, which is a risk factor for noncommunicable diseases [14].

As performed in previous studies [14–16] and grounded on previous literature [14, 18, 31, 32, 41–45], each dimension was operationalized using different measurements derived from accelerometry or questionnaires (Table 1). The metrics were subsequently dichotomized based on thresholds recommended from guidelines and consistent with prior studies investigating multidimensional sleep health assessment (Table 1) [14, 18, 31, 32, 41–43, 46, 47]. To each dimension was assigned a score of 0 (poor sleep) or 1 (good sleep), and the scores from the six different sleep measures were summed, resulting in a potential 0–6 range with an equal weighting of each sleep dimension. Higher scores indicate greater sleep health.

**Table 1:** Sleep dimensions included in the sleep health index.

Sleep dimension	Measurement	Cut-off for healthy sleep	**Reference** ^a^ ^,^ ^b^
Efficiency	Sleep efficiency	>=85%	Schutte-Rodin et al. 2008; Lee et al. 2023; Bruno et al. 2024
Duration	Total sleep time	7-9 h	Mukherjee et al. 2015; Bruno et al. 2024
Alertness	Epworth sleepiness scale	<=10	Vignatelli, 2003; Bruno et al. 2024
Regularity	Sleep regularity index	>=45.8	Phillips et al. 2017; Bruno et al. 2024
Timing	Sleep midpoint	02:00–04:00	Buysse et al. 2014; Bruno et al. 2024
Satisfaction	Pittsburgh sleep quality index	<=5	Curcio, 2013; Bruno et al. 2024

a Sleep efficiency, total sleep time, the sleep regularity index and sleep midpoint were derived from accelerometry.

b For each dimension the metric used for quantification, the cut-off to identify healthy and unhealthy sleep, and the reference justifying the choice of the cut-off are provided. Complete citation details are available in the Reference section of the Main Text.

### Data analysis

Data were analyzed in RStudio 4.4.1. Mean (M) and standard deviation (SD) described quantitative variables, a table of frequencies, and percentages presented categorical variables.

First, parametric tests were used to test possible differences in sleep and circadian parameters between SjD patients and HCs. Multiple linear regression models were subsequently estimated to confirm the results from parametric tests, including age and BMI as possible confounding factors [14, 18, 22–24]. Sex was excluded from possible confounding factors as our sample only included female participants.

Moreover, accelerometric data were analysed through functional linear modelling (FLM), a statistical framework specifically developed for the analysis of accelerometric time series [48]. This method was applied to explore possible between-group differences in accelerometric data as a function of time, possibly demonstrating diverging circadian patterns of activity [48, 49]. A detailed description of FLM application to gathered accelerometric data is available in Supplementary Data S2.

In SjD patients, multiple linear regression models were finally fitted to explore possible associations between sleep and circadian parameters (i.e. the dependent variables), and the following independent variables: disease activity, patient-reported SjD symptoms severity (i.e. ESSPRI), severity of anxiety and depression symptoms, and possible confounding variables. Fatigue severity, as measured by the FACIT scale, was not included among independent predictors of sleep and circadian parameters to avoid multicollinearity of the regression models. As fatigue severity is an item of the ESSPRI, a significant correlation between ESSPRI and FACIT was indeed expected.

As the treatment for mood disorders and/or insomnia may impact sleep and circadian parameters [22–24], we conducted a sensitivity analysis by fitting the aforementioned regression models only in SjD patients untreated for these conditions at recruitment [22–24]. The significance level was set at *P* < 0.05. An a priori power analysis yielded a minimum sample size of 39 per group (α = 0.05, β = 95%, Supplementary Data S3).

## Results

### Sociodemographic and clinical data

Forty-six Sjögren Disease (SjD) patients and 40 age-, BMI-, and sex-matched healthy controls (HCs) were enrolled in the study (Table 2). No shift workers were included in the study. In SjD patients, the mean ESSDAI was 2.61 (3.34), with 8 patients being classified as active (17%). Based on ESSPRI scores (*M* = 6.15; SD = 2.51), 33 patients showed an unsatisfactory symptom state (72%). SjD patients’ clinical data is shown in Table 2.

**Table 2:** Participants’ sociodemographic and clinical data.

**Variables** ^a^	**SjD Patients** *N* = 46^b^	**HCs** *N* = 40^b^	***P*-value** ^c^
Age	56 (13)	54 (9)	0.4
BMI	23.28 (3.37)	23.33 (3.63)	>0.9
Females	46 (100%)	40 (100%)	>0.9
Caucasian ethnicity	46 (100%)	40 (100%)	>0.9
Age at diagnosis	48 (12)		
Disease duration	9 (7)		
**Clinical manifestations at the time of recruitment**			
ESSDAI	2.57 (3.36)		
Active disease	8 (17%)		
ESSPRI	6.15 (2.51)		
Unsatisfactory symptom state	33 (72%)		
ESSPRI dryness	6.67 (2.55)		
ESSPRI fatigue	6.24 (2.85)		
ESSPRI pain	5.5 (3.2)		
Fibromyalgia	12 (26%)		
HADS	15 (7.0)		
FACIT	34 (12)		
Hypertension	12 (26%)		
Non-Hodgkin lymphoma	2 (4.3%)		
Lymphadenopathy	6 (13%)		
Glandular	3 (6.5%)		
Articular	6 (13%)		
Pulmonary	1 (2.2%)		
Renal	2 (4.3%)		
Hematological	10 (22%)		
Antibodies			
SSA	35 (76%)		
SSB	18 (40%)		
Ro52	32 (70%)		
Ro60	27 (59%)		
**Treatment at the time of recruitment**			
Hydroxychloroquine	21 (47%)		
Biological treatment	22 (48%)		
Glucocorticoids	3 (6.7%)		
Treatment for mood disorders and/or insomnia	6 (6.7%)		

a SjD: Sjögren’s Disease; HCs: healthy controls; ESSDAI: EULAR Sjögren’s Syndrome Disease Activity Index; ESSPRI: EULAR Sjogren’s Syndrome Patient Reported Index; HADS: Hospital Anxiety and Depression Scale; FACIT: Functional Assessment of Chronic Illness Therapy-Fatigue.

b Mean (SD); n (%).

c Welch Two Sample *t* test for continue variables, Chi-squared test for categorical variables.

### Differences in sleep and circadian parameters between sjögren disease patients and healthy controls

SjD patients showed significantly reduced sleep health (M = 3.59; SD = 1) compared with HCs (*M* = 4.2, SD = 0.97, *P* = 0.005; Table 3), due to lower reported sleep quality (PSQI, *P* < 0.001), higher sleepiness (ESS, *P* < 0.046), and increased accelerometric sleep fragmentation, as assessed through SE (*P* < 0.001) and WASO (*P* < 0.001). Moreover, significant later mid-sleep point (*P* = 0.009), acrophase (*P* = 0.033), self-reported wake-up time (*P* < 0.001), and sleep offset (*P*  < 0.001), were observed in SjD patients, suggesting the presence of a delay in SjD patients’ circadian activity patterns compared with controls.

**Table 3:** Differences in sleep and circadian parameters between Sjögren disease patients and healthy controls.

**Sleep parameters** ^a^	**SjD patients** *N* = 46^b^	**HCs** *N* = 40^b^	***P*-value** ^c^
Sleep Health Index	3.59 (1.00)	4.20 (0.97)	**0.005**
Sleep quality (PSQI)	9.6 (3.6)	7.2 (2.8)	**<0.001**
TST	7.14 (1.58)	7.04 (1.27)	0.8
WASO (min)	94 (44)	59 (33)	**<0.001**
SE	82 (10)	88 (6)	**<0.001**
SRI	78 (12)	79 (10)	0.8
Bedtime	22:52 (87 min)	22:52 (62 min)	>0.9
Wake-up time	07:46 (72 min)	06:58 (51 min)	**<0.001**
Sleep onset	23:02 (79 min)	22:56 (84 min)	0.7
Sleep offset	07:14 (57 min)	06:35 (39 min)	**<0.001**
Mid-sleep point	03:24 (63 min)	02:50 (53 min)	**0.009**
rMEQ	16.9 (3.5)	17.4 (3.7)	0.5
ESS	8.1 (4.4)	6.4 (3.1)	**0.046**
Daily steps	10,008 (4,697)	11,461 (4,751)	0.2
Acrophase	15:25 (67 min)	14:56 (52 min)	**0.033**
MESOR	0.59 (0.05)	0.60 (0.06)	0.5
Amplitude	0.40 (0.06)	0.39 (0.08)	0.3
IS	0.83 (0.11)	0.83 (0.08)	>0.9
IV	0.31 (0.08)	0.31 (0.06)	0.7
RA	0.86 (0.12)	0.84 (0.19)	0.6

Bold text highlights *P*-values <0.05.

a SjD: Sjögren’s Disease; HCs: healthy controls; PSQI: Pittsburgh Sleep Quality Index; TST: Total Sleep Time (h); WASO: Wake After Sleep Onset (min); SE: Sleep Efficiency (%); Sleep Regularity Index (%); rMEQ: reduced Morningness-Eveningness Questionnaire); ESS: Epworth Sleepiness Scale; MESOR: Midline-Estimating Statistic Of Rhythm; IS: Interdaily Stability; IV: Interdaily Variability; RA: Relative Amplitude.

b Mean (SD); n (%);.

c Welch Two Sample *t* test for continue variables, Chi-squared test for categorical variables.

Multiple linear regression models confirmed the results from parametric tests, adjusting for age and BMI (Table 4). From a quantitative standpoint, SjD patients showed a sleep health index that was, on average, 0.61 points lower than that of HCs (*P* = 0.006) indicating poorer sleep health, along with an estimated 33-min longer duration of awakenings (WASO, *P* < 0.001) and an average increase of 2.4 and 1.8 points in PSQI (*P* = 0.001) and ESS scores (*P* = 0.040), respectively. Moreover, SjD patients’ mid-sleep point was delayed by an average of 35 min (*P* = 0.008) compared with HCs. Coherently, an average 29-min later acrophase was observed in SjD patients (*P* = 0.029).

**Table 4:** Linear regression models investigating possible associations between SjD patients and sleep and circadian parameters.

**Independent variables** ^a^	**SjD patients** β (*P*)	**Age** β (*P*)	**BMI** β (*P*)	R2	Adjusted R2
Sleep Health Index	−0.61 (**0.006**)	0.00 (0.9)	−0.02 (0.4)	0.097	0.063
PSQI	2.4 (**0.001**)	0.03 (0.4)	0.04 (0.7)	0.133	0.102
ESS	1.8 (**0.040**)	−0.04 (0.3)	0.13 (0.3)	0.072	0.038
TST	0.06 (0.9)	0.02 (0.2)	0.02 (0.7)	0.027	−0.009
SE	−6.2 (**0.001**)	−0.11 (0.2)	−0.12 (0.6)	0.15	0.119
WASO	33 (**<0.001**)	0.94 (**0.016**)	0.58 (0.6)	0.229	0.201
SRI	−0.97 (0.7)	0.14 (0.2)	−0.34 (0.3)	0.03	−0.005
Mid-sleep point	0.58 (**0.008**)	−0.01 (0.3)	0.00 (>0.9)	0.088	0.055
rMEQ	−0.67 (0.4)	0.08 (**0.028**)	−0.02 (0.9)	0.063	0.028
Daily Steps	−1,576 (0.12)	54 (0.2)	−301 (**0.041**)	0.084	0.051
Acrophase	29 (**0.029**)	−0.01 (0.3)	0.02 (0.6)	0.068	0.034
Amplitude	0.02 (0.3)	0.00 (0.14)	0.00 (0.2)	0.059	0.025
MESOR	−0.01 (0.5)	0.00 (0.7)	0.00 (0.7)	0.008	−0.028
IS	0.00 (0.9)	0.003 (**0.007**)	0.00 (0.4)	0.092	0.059
IV	0.01 (0.7)	0.00 (>0.9)	0.00 (0.3)	0.015	−0.021
RA	0.02 (0.6)	0.00 (0.4)	−0.01 (0.2)	0.033	−0.003

Bold text highlights *P*-values <0.05.

a SjD: Sjögren’s Disease; BMI: Body-Mass Index (kg/m^2^); PSQI: Pittsburgh Sleep Quality Index; TST: Total Sleep Time (h); WASO: Wake After Sleep Onset (min); SE: Sleep Efficiency (%); Sleep Regularity Index (%); rMEQ: reduced Morningness-Eveningness Questionnaire); ESS: Epworth Sleepiness Scale; MESOR: Midline-Estimating Statistic Of Rhythm; IS: Interdaily Stability; IV: Interdaily Variability; RA: Relative Amplitude.

### Circadian patterns of accelerometric data in sjögren disease patients and healthy controls

**Figure 1:**
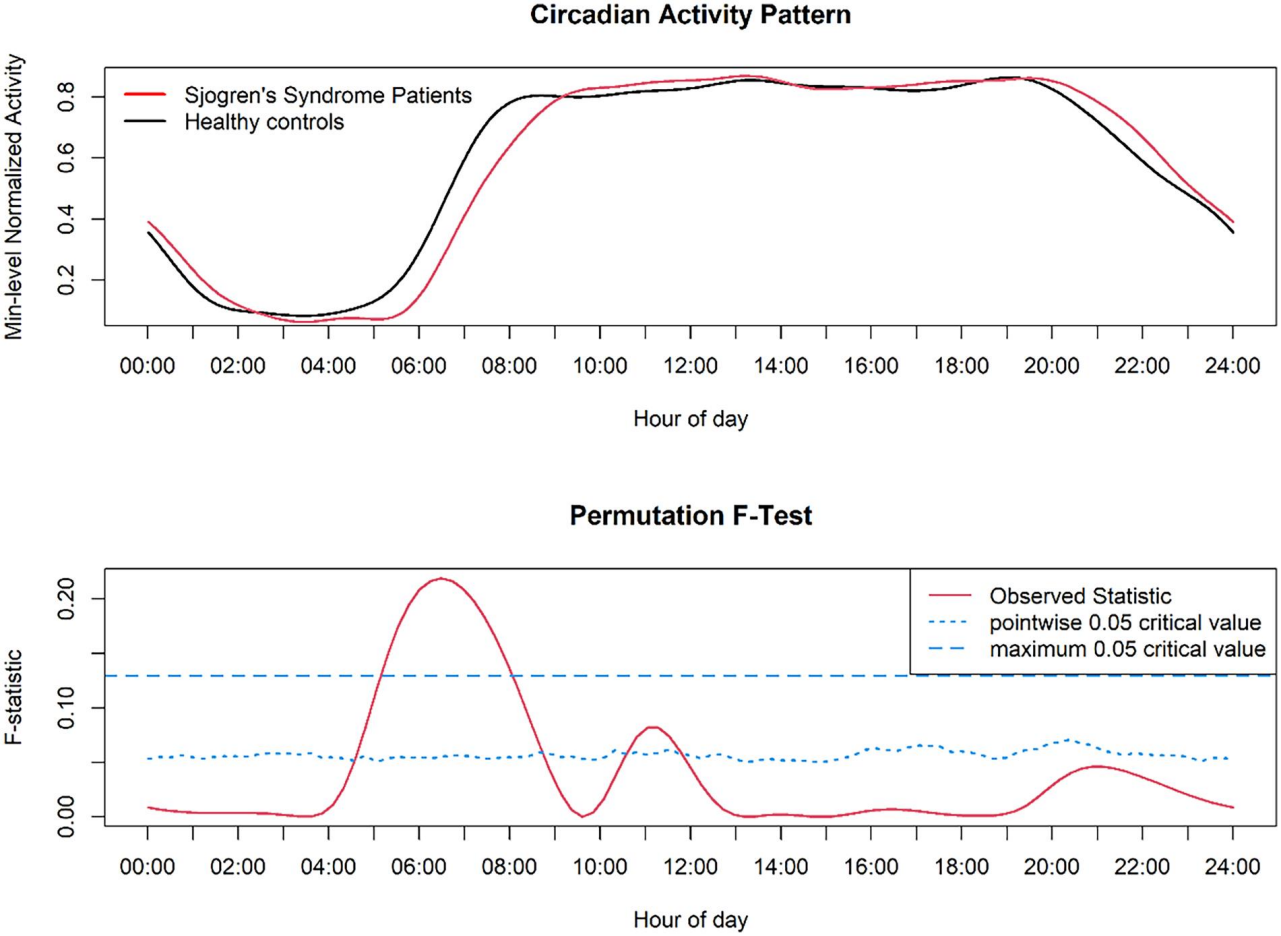
Circadian activity pattern in Sjögren disease patients and healthy controls. In the overhead panel, minute-level normalized Activity Units are shown. In the panel showing the nonparametric permutation *F*-tests, the continuous red solid line corresponds to the observed statistics, the dashed blue line represents the global test of significance, and the dotted blue line represents the less conservative pointwise test of significance. Significant differences are observed whenever the observed statistic is above the pointwise or global threshold of significance. Min: minute

To explore possible between-group differences in accelerometric data across the 24-h, functional linear modelling was employed (Fig. 1).

The *F*-test showed significantly lower activity in SjD patients during the 04:30–08:30 time frame compared with HCs, as measured by accelerometry. Conversely, significantly higher activity was observed in SjD patients between 10:30 and 11:30 compared with HCs.

### Linear regression models investigating possible predictors of sleep and circadian parameters in sjögren disease patients

Multiple linear regression models were fitted in the SjD group to explore possible associations between sleep and circadian parameters (i.e. the dependent variables), and the following independent variables: disease activity, patients’ symptoms burden, severity of anxiety and depression symptoms, and possible confounding variables (Table 5). The FACIT scale was not included among independent predictors to avoid multicollinearity of the regression models. A Pearson’s correlation test indeed confirmed the significant correlation between FACIT and ESSPRI (*r*=−0.62; *P* < 0.001).

**Table 5:** Linear regression models investigating possible predictors of sleep and circadian parameters in SjD patients.

**Independent variables** ^a^	**ESSDAI** β (*P*)	**ESSPRI** β (*P*)	**HADS** β (*P*)	**Age** β (*P*)	**BMI** β (*P*)	R2	Adjusted R2
Sleep Health Index	0.00 (0.6)	−0.04 (0.6)	−0.03 (0.2)	0.00 (>0.9)	−0.07 (0.2)	0.127	0.015
PSQI	0.06 (0.6)	0.41 (**0.044**)	0.25 (**<0.001**)	−0.02 (0.5)	−0.16 (0.2)	0.51	0.447
ESS	0.04 (0.8)	0.83 (**0.010**)	−0.13 (0.2)	−0.07 (0.2)	0.26 (0.2)	0.209	0.107
TST	0.10 (0.13)	0.08 (0.5)	−0.08 (**0.035**)	0.03 (0.13)	0.00 (>0.9)	0.199	0.096
SE	0.08 (0.9)	0.96 (0.2)	−0.65 (**0.012**)	−0.07 (0.6)	−0.17 (0.7)	0.169	0.062
WASO	−0.07 (>0.9)	−4.4 (0.2)	2.3 (**0.043**)	0.87 (0.11)	0.58 (0.8)	0.163	0.056
SRI	−0.77 (0.2)	−0.89 (0.3)	0.48 (0.12)	−0.03 (0.9)	−0.36 (0.5)	0.126	0.014
Midpoint	−0.02 (0.7)	−0.01 (>0.9)	0.00 (>0.9)	0.00 (>0.9)	−0.01 (0.8)	0.007	−0.121
rMEQ	0.26 (0.11)	−0.27 (0.3)	0.01 (>0.9)	0.03 (0.4)	0.14 (0.4)	0.124	0.012
Daily Steps	−526 (**0.013**)	310 (0.4)	−238 (**0.041**)	27 (0.6)	−48 (0.8)	0.218	0.118
Acrophase	−0.01 (0.8)	0.03 (0.7)	−0.05 (0.073)	0.00 (0.9)	−0.02 (0.7)	0.1	−0.015
Amplitude	0.00 (0.7)	0.00 (0.7)	0.00 (0.5)	0.00 (0.5)	0.00 (0.5)	0.068	−0.051
MESOR	−0.01 (**0.007**)	0.00 (>0.9)	0.00 (0.9)	0.00 (>0.9)	0.00 (0.7)	0.171	0.065
IS	0.00 (0.6)	−0.01 (0.2)	0.00 (0.10)	0.00 (0.14)	0.00 (0.7)	0.133	0.022
IV	0.00 (0.4)	−0.01 (0.3)	0.00 (0.5)	0.00 (0.4)	0.00 (0.8)	0.096	−0.019
RA	0.00 (0.9)	0.01 (0.5)	0.00 (0.8)	0.00 (0.5)	0.00 (0.5)	0.042	−0.08

Bold text highlights *P*-values <0.05.

a ESSDAI: EULAR Sjögren’s Syndrome Disease Activity Index; ESSPRI: EULAR Sjogren’s Syndrome Patient Reported Index; HADS: Hospital Anxiety and Depression Scale; BMI: Body-Mass Index (kg/m^2^); PSQI: Pittsburgh Sleep Quality Index; TST: Total Sleep Time (h); WASO: Wake After Sleep Onset (min); SE: Sleep Efficiency (%); Sleep Regularity Index (%); rMEQ: reduced Morningness-Eveningness Questionnaire); ESS: Epworth Sleepiness Scale; MESOR: Midline-Estimating Statistic Of Rhythm; IS: Interdaily Stability; IV: Interdaily Variability; RA: Relative Amplitude.

In these models, perceived sleep quality (PSQI, β = 0.41, *P* = 0.044) and sleepiness (ESS, β = 0.83 *P* = 0.010) were significantly predicted by patient-reported SjD symptoms severity, as measured by ESSPRI. Differently, disease activity (i.e. ESSDAI) significantly predicted the number of daily steps (β=-526, *P* = 0.013) and MESOR (β=−0.01, *P* = 0.007). Finally, severity of anxiety and depression significantly predicted PSQI (β = 0.25, *P* < 0.001), sleep duration (TST, β=−0.08, *P* = 0.035), sleep fragmentation (SE, β=−0.65, *P* = 0.012; WASO, β = 2.3, *P* = 0.043), and number of daily steps (β=−238, *P* = 0.041).

When excluding SjD patients receiving treatment for mood disorders and/or insomnia, the aforementioned results were confirmed (Supplementary Table S1). Moreover, disease activity emerged as a significant predictor of both sleep duration (β = 0.15, *P* = 0.0496) and regularity (SRI, β=−1.3, *P* = 0.030).

## Discussion

This study offers novel insights into the interplay between sleep, circadian rhythms, and clinical outcomes in patients with Sjögren Disease (SjD). In detail, our findings demonstrate that SjD patients show impaired sleep parameters, sleep health, and circadian rhythms compared with healthy controls (HCs), as assessed by both subjective and objective methods. Importantly, such disruptions were differentially associated with disease activity and patient-reported symptoms, underscoring the multifaceted nature of sleep disturbances in these patients. To our knowledge, this is the first study assessing sleep health, rest-activity and circadian rhythms in patients with SjD.

Compared with HCs, patients with SjD reported significantly worse sleep quality and greater daytime sleepiness [2, 4, 9]. In parallel, higher accelerometric sleep fragmentation was observed in patients with SjD. While these findings are consistent with previous sleep laboratory studies reporting impaired subjective and objective sleep parameters in patients with SjD [6, 10], they first confirm that such disturbances persist in ecological settings. The assessment of a wide range of objective and subjective sleep parameters allowed for the estimation of a multidimensional Sleep Health Index, based on the RU-SATED framework. Our analyses indeed demonstrated that patients with SjD show an average 0.61 increase in the number of impaired sleep health dimensions compared with HCs (standard error 0.21). Previous literature demonstrated that a reduction in a single component of RE-SATED sleep health is associated with an average 27% increase in all-cause mortality [19], an average 141% increase in the odds of suffering from heart diseases [16], and an average 11% increase in cardiometabolic morbidity [15]. Given such established link between impaired sleep health and adverse health outcomes [18], our findings may suggest relevant prognostic implications for patients with SjD, which should be investigated by further research. Moreover, a 0.61 between-group difference in the Sleep Health Index represents a 10% shift across the entire scale range. Such percentage change aligns with previously reported minimally clinical important differences for other widely used tools, e.g. ISI, PSQI, and VAS for sleep quality, ranging from 8.6% to 14% [44, 50, 51]. Altogether, these findings highlight the possible relevance of sleep disturbances in the clinical picture of SjD patients. Consequently, prioritizing the screening and management of this underrecognized component of the disease burden is currently needed.

Our study firstly investigated rest-activity circadian rhythms in SjD. Specifically, we observed significant delays in SjD patients’ sleep midpoint and acrophase, suggesting an objective shift toward eveningness compared with HCs. This circadian phase delay was paralleled by time-specific differences in 24-h accelerometric patterns, with SjD patients displaying reducedearly-morning activity compared with HCs, reflecting later awakening times. Notably, such objective shift in the rest-activity rhythms was not reflected by a significant between-group difference in self-reported circadian preferences, as assessed by the rMEQ. This discrepancy may be primarily due to a behavioural adaptation to impaired sleep continuity in SjD patients. Compared with HCs, SjD patients indeed showed higher sleep fragmentation as measured by an average 33-min increase in WASO (*P* < 0.001), with no significant differences in sleep duration and sleep onset time being observed. Along with the described delay in sleep offset and wake-up time, these findings suggest that SjD patients’ objective delay rest-activity rhythms may be driven by prolonged wake during bedtime, with no differences in circadian preferences compared with HCs. This interpretation aligns with the observed impairments in self-reported sleep quality and sleepiness in SjD patients.

In parallel, this study identifies possible clinical correlates of sleep disturbances and accelerometric movement measures, i.e. MESOR and daily steps. Subjective sleep quality and daytime sleepiness were indeed significantly associated with higher anxiety and depression severity and greater symptoms burden, as measured by the ESSPRI. These findings support the growing recognition that non-immunologic factors, including mood and psychological distress, may play a significant role in the pathophysiology of fatigue and poor sleep in SjD [2, 5, 8]. Conversely, objective movement measures were associated with disease activity irrespective of both reported symptoms burden and mood symptoms, suggesting that immune-mediated processes affect daytime functioning. Moreover, increased disease activity predicted increased total sleep time and reduced sleep regularity, aligning with previous data in other rheumatologic populations [22]. Systemic inflammation, pain-related sleep continuity disruption, and the aforementioned reduction in activity may be responsible for such sleep disturbances [21, 52]. These data suggest how psychological and immunological pathways may shape partially divergent sleep and circadian disturbances in SjD, possibly requiring different managements. Moreover, these findings pave the way towards the implementation of a multidimensional sleep characterization into clinical practice, allowing for the recognition of different sleep disruption patterns.

Although the absence of sleep diaries administration is a major limitation of our study, retrospectively reported bedtime and wake-up time were assessed via the PSQI (Table 3). On average, the sleep episodes detected by accelerometry were included within the self-reported time in bed, that is, the time between self-reported bedtime and wake-up time (Table 3). Notably, this concordance possibly supports the accuracy of sleep episodes detection by our 7-day accelerometric monitoring. Nevertheless, the impossibility to perform a day-by-day comparison between accelerometric and self-report data and the partial disagreement between the time windows investigated by accelerometry and the PSQI, i.e., 1 week and 1 month, respectively, warrant caution in the interpretation of our data.

The employment of normalized raw data from consumer-grade accelerometers may have limited the accuracy of sleep fragmentation and circadian rhythm metrics estimation in our study, possibly impacting the robustness and generalizability of our findings. Specifically, low specificity in sleep-wake detection was previously observed when analyzing such raw data through the Fitbit proprietary algorithm or widely-used, open-source algorithms [53]. Nonetheless, Fitbit raw data were analyzed using a validated, certified, medical-grade algorithm for sleep–wake classification in this work. This artificial neural network was previously validated against polysomnography [34], showing strong agreement in sleep metrics estimation. Furthermore, the employment of popular algorithms to estimate circadian rhythm metrics from Fitbit raw accelerometric data, e.g. cosinor analysis, has been validated against their application on research-grade raw accelerometric data [54, 55]. Finally, both the DORMI algorithm and circadian rhythm analyses have been previously applied to Fitbit raw data from multiple cohorts, including rheumatologic populations [14, 22–24]. Altogether, such data may partially limit the constraints related to the employment of a consumer-grade device in our work.

Albeit the dichotomization of continuous sleep variables may result in loss of information and limit the interpretability of the constructed Sleep Health Index, we conducted separate analyses for each sleep dimension of the index (Tables 3–5), to preserve the information inherent to continuous variables. Moreover, the cross-sectional design of this study limits causal inferences binding clinical outcomes and sleep and circadian disturbances and the exclusively female sample limits generalizability to males, though it represented the epidemiology of the disease [1]. Lastly, our sample of SjD patients included a relatively low number of active patients as assessed by the ESSDAI, which may depend on the timing of the recruitment phase (i.e. outpatient visits), when disease activity is generally better controlled in comparison to inpatient settings. Further research should aim at confirming these results in SjD cohorts including a larger number of active patients.

In conclusion, our study demonstrated that patients with SjD show impaired sleep parameters, sleep health, and circadian rhythms compared with healthy controls, with different clinical outcomes possibly shaping partially diverging sleep and circadian disruption patterns in patients with SjD. These findings emphasize the necessity of a multidimensional approach to sleep and circadian rhythms characterization in rheumatologic populations [22–24], and its possible application in both research and clinical practice.

## Supplementary Material

keag134_Supplementary_Data

## Data Availability

The data underlying this article will be shared on reasonable request to the corresponding author. The administration of the FACIT scale was conducted according to the license from the copyright holders.
