# Supplementary material for: Exploring sleep health and circadian rhythm disruption in Sjögren’s disease: an accelerometric and self-reported cross-sectional study
Source: Rheumatology (Oxford). 2026 Mar 21;65(4):keag134. doi: 10.1093/rheumatology/keag134 (PMC13061132; doi:10.1093/rheumatology/keag134)
Supplement: keag134_Supplementary_Data [file keag134_supplementary_data.docx]

# Supplementary Data

## Supplementary Data S1: employed Self-report Questionnaires

The EULAR primary Sjögren’s syndrome patient reported index (ESSPRI) is a self-report questionnaire investigating the severity of SjD-related symptoms over the past 2 weeks (1). The ESSPRI is composed of 3 items, i.e., dryness, fatigue, and limb pain, each one scored on a 0-10 numerical rating scale, with the ESSPRI total score being calculated as the average of the items’ scores (1,2). An unsatisfactory symptom state was defined as ESSPRI≥5 (3). The Hospital Anxiety and Depression Scale (HADS) is a 14-items self-administered questionnaire assessing the severity of anxiety and depression symptoms (4,5). The HASD is composed of 7 anxiety-related items (HADS-A subscale) and 7 depression-related items (HADS-D subscale), with each item being rated on a score ranging from 0 to 3 (4,5). The sum of the scores ranges from 0 to 42, with higher scores suggesting more severe symptoms (4,5). The Functional Assessment of Chronic Illness Therapy-Fatigue (FACIT) is a widely used, 13-item patient-reported outcome measure to assess the severity of fatigue (6). Patients rate each item on a 5-point Likert scale and the individual item scores are summed to create a total score ranging from 0 to 52 (6). Higher scores on the FACIT-Fatigue scale indicate lower fatigue severity (6).

The PSQI is a self-administered 19-item questionnaire measuring sleep quality over a one-month time interval (7). The sum of the score ranges from 0 to 21; a global score >5 is an index of poor sleep quality, defining an individual as a poor sleeper (7). Daytime sleepiness was measured through the Epworth Sleepiness Scale (ESS), a widely used self-reported questionnaires composed of 8 items (8,9). The sum of the score ranges from 0 to 24, with a global score higher than 10 indicating excessive daytime sleepiness (8,9). The reduced Morningness-Eveningness Questionnaire (rMEQ) is the reduced version of the MEQ (10), a self-administered questionnaire to estimate the chronotype of an individual, validated in Italian language (11). The total score ranges from 4 to 26: according to the scale, a score <11 indicates an evening type; 11–18 a neither type; >18 a morning type.

## Supplementary Data S2: Functional Linear Modelling

Functional linear modelling (FLM) was applied to explore possible between-group differences in actigraphic data as a function of time, possibly demonstrating diverging circadian patterns of activity. Operationally, FLM involves two steps: first, a basis function model converts the raw actigraphic data into a functional form that captures the major trend and reduces data variability. Based on previous literature (12–14), functional smoothing was performed via a Fourier expansion model with n=9 basis functions. Second, sets of functions are compared using a nonparametric permutation F-test, which derives a theoretical null distribution by randomly rearranging group assignments (i.e., SjD group and HCs group) (12–14). Both pointwise (proportion of 500 permutation F-values at each time point) and global (maximized F-value from each permutation) tests of significance were performed (12–14).

## Supplementary Data S3: Power Analysis

Given the absence of previous studies investigating circadian parameters in SjD patients, an *a priori* power analysis was performed based on the study from Miyauchi et al. (15), which provides the only available comparison of both self-report and actigraphic sleep parameters between SjD patients and HCs. Moreover, in light of the exploratory nature of our research, we aimed at estimating the minimum sample size required to detect at least one significant between-group difference in such parameters. Consequently, we derived Cohen’s d effect sizes from the parametric tests reported by Miyauchi et al, and subsequently employed the maximum observed effect size in our power analysis (Cohen’s d = 0.76), corresponding to the magnitude of the association between PSQI and the group variable (SjD patients vs HCs). Based on this and considered the partial limitations of our *a priori* assumptions, we finalized the power analysis with a 0.05 significance level and a conservative 95% power, yielding a minimum sample size of 39 per group. The power analysis was conducted using the GPower 3.1.9.7 Software.

# Supplementary Tables

## Supplementary Table S1

| **SUPPLEMENTARY TABLE S1: Linear Regression Models Investigating Possible Predictors of Sleep and Circadian Parameters in SjD patients, excluding SjD patients assuming treatment for mood disorders and/or insomnia (N=40).** | | | | | | | |  |
| --- | --- | --- | --- | --- | --- | --- | --- | --- |
| Independent Variables | ESSDAI | ESSPRI | HADS | Age | BMI | R2 | Adjusted R2 |  |
| Sleep Health Index | -0.01 (0.8) | -0.02 (0.8) | -0.04 (0.2) | -0.01 (0.5) | -0.10 (**0.028**) | 0.236 | 0.120 |  |
| PSQI | -0.03 (0.8) | 0.51 (**0.024**) | 0.23 (**0.003**) | -0.05 (0.2) | -0.25 (0.054) | 0.572 | 0.507 |  |
| ESS | 0.13 (0.5) | 0.54 (0.13) | -0.07 (0.6) | -0.04 (0.5) | 0.42 (**0.046**) | 0.212 | 0.093 |  |
| TST | 0.15 (**0.050**) | -0.03 (0.8) | -0.05 (0.2) | 0.03 (0.2) | 0.01 (0.9) | 0.215 | 0.096 |  |
| SE | 0.20 (0.7) | 0.73 (0.3) | -0.53 (**0.030**) | -0.13 (0.3) | -0.18 (0.7) | 0.206 | 0.085 |  |
| WASO | -0.06 (>0.9) | -4.3 (0.2) | 2.1 (0.068) | 1.2 (**0.038**) | 0.87 (0.7) | 0.218 | 0.1 |  |
| SRI | -1.3 (**0.030**) | 0.37 (0.7) | 0.15 (0.6) | -0.12 (0.4) | -0.79 (0.2) | 0.219 | 0.101 |  |
| Midpoint | -0.01 (>0.9) | -0.04 (0.7) | 0.01 (0.8) | 0.00 (>0.9) | 0.00 (>0.9) | 0.005 | -0.146 |  |
| rMEQ | 0.27 (0.13) | -0.38 (0.2) | 0.01 (>0.9) | 0.06 (0.2) | 0.21 (0.2) | 0.166 | 0.039 |  |
| Daily Steps | -558 (**0.020**) | 467 (0.2) | -278 (**0.031**) | 12 (0.8) | -151 (0.5) | 0.231 | 0.114 |  |
| Acrophase | 0.02 (0.7) | -0.01 (>0.9) | -0.04 (0.3) | 0.00 (>0.9) | -0.01 (0.9) | 0.078 | -0.062 |  |
| Amplitude | 0.00 (0.5) | 0.00 (0.6) | 0.00 (0.8) | 0.00 (0.6) | 0.00 (0.7) | 0.066 | -0.075 |  |
| MESOR | -0.01 (**0.008**) | 0.00 (0.5) | 0.00 (0.4) | 0.00 (0.8) | 0.00 (0.8) | 0.199 | 0.078 |  |
| IS | 0.00 (0.5) | -0.01 (0.5) | 0.00 (0.3) | 0.00 (0.2) | 0.00 (0.9) | 0.113 | -0.022 |  |
| IV | 0.01 (0.11) | -0.01 (0.074) | 0.00 (0.8) | 0.00 (0.2) | 0.00 (0.8) | 0.181 | 0.057 |  |
| RA | 0.00 (0.9) | 0.01 (0.6) | 0.00 (0.9) | 0.00 (0.6) | 0.00 (0.5) | 0.04 | -0.105 |  |
| ^1^ESSDAI: EULAR Sjögren’s Syndrome Disease Activity Index; ESSPRI: EULAR Sjogren's Syndrome Patient Reported Index; HADS: Hospital Anxiety and Depression Scale;​ BMI: Body-Mass Index (kg/m^2^); PSQI: Pittsburgh Sleep Quality Index; TST: Total Sleep Time (hours); WASO: Wake After Sleep Onset (minutes); SE: Sleep Efficiency (%); Sleep Regularity Index (%); rMEQ: reduced Morningness-Eveningness Questionnaire); ESS: Epworth Sleepiness Scale; MESOR: Midline-Estimating Statistic Of Rhythm; IS: Interdaily Stability; IV: Interdaily Variability; RA: Relative Amplitude. | | | | | | | | |

# Supplementary Data References

1. Seror R, Theander E, Brun JG, Ramos-Casals M, Valim V, Dörner T, et al. Validation of EULAR primary Sjögren’s syndrome disease activity (ESSDAI) and patient indexes (ESSPRI). Ann Rheum Dis. 2015 May 1;74(5):859–66.

2. Seror R, Ravaud P, Mariette X, Bootsma H, Theander E, Hansen A, et al. EULAR Sjogren’s Syndrome Patient Reported Index (ESSPRI): development of a consensus patient index for primary Sjogren’s syndrome. Ann Rheum Dis [Internet]. 2011 Jun [cited 2025 May 30];70(6):968–72. Available from: https://pubmed.ncbi.nlm.nih.gov/21345815/

3. Seror R, Bootsma H, Saraux A, Bowman SJ, Theander E, Brun JG, et al. Defining disease activity states and clinically meaningful improvement in primary Sjögren’s syndrome with EULAR primary Sjögren’s syndrome disease activity (ESSDAI) and patient-reported indexes (ESSPRI). Ann Rheum Dis [Internet]. 2016 Feb 1 [cited 2025 May 30];75(2):382–9. Available from: https://pubmed.ncbi.nlm.nih.gov/25480887/

4. Zigmond AS, Snaith RP. The Hospital Anxiety and Depression Scale. Acta Psychiatr Scand [Internet]. 1983 Jun 1 [cited 2025 May 30];67(6):361–70. Available from: https://onlinelibrary.wiley.com/doi/full/10.1111/j.1600-0447.1983.tb09716.x

5. Costantini M, Musso M, Viterbori P, Bonci F, Del Mastro L, Garrone O, et al. Detecting psychological distress in cancer patients: Validity of the Italian version of the Hospital Anxiety and Depression Scale. Supportive Care in Cancer [Internet]. 1999 May [cited 2025 May 30];7(3):121–7. Available from: https://link.springer.com/article/10.1007/s005200050241

6. Griffiths N, Wratten S, Flynn J, Bookman AAM, Ndife B, Hueber W, et al. Content Validity of Sjögren’s Syndrome Symptom Diary and Functional Assessment of Chronic Illness Therapy-Fatigue in Patients with Sjögren’s. Rheumatol Ther [Internet]. 2022 Dec 1 [cited 2025 Jun 2];9(6):1559–74. Available from: https://link.springer.com/article/10.1007/s40744-022-00489-y

7. Curcio G, Tempesta D, Scarlata S, Marzano C, Moroni F, Rossini PM, et al. Validity of the Italian Version of the Pittsburgh Sleep Quality Index (PSQI). Neurological Sciences [Internet]. 2013 Apr 13;34(4):511–9. Available from: http://link.springer.com/10.1007/s10072-012-1085-y

8. Vignatelli L, Plazzi G, Barbato A, Ferini-Strambi L, Manni R, Pompei F, et al. Italian version of the Epworth sleepiness scale: External validity. Neurological Sciences [Internet]. 2003 Feb [cited 2023 Aug 24];23(6):295–300. Available from: https://link.springer.com/article/10.1007/s100720300004

9. Johns MW. A New Method for Measuring Daytime Sleepiness: The Epworth Sleepiness Scale. Sleep [Internet]. 1991 Nov 1 [cited 2023 Aug 24];14(6):540–5. Available from: https://dx.doi.org/10.1093/sleep/14.6.540

10. Adan A, Almirall H. Horne &amp; Östberg morningness-eveningness questionnaire: A reduced scale. Pers Individ Dif [Internet]. 1991 [cited 2022 Sep 12];12(3):241–53. Available from: https://linkinghub.elsevier.com/retrieve/pii/019188699190110W

11. Natale V, Esposito MJ, Martoni M, Fabbri M. Validity of the reduced version of the Morningness-Eveningness Questionnaire. Sleep Biol Rhythms. 2006 Feb;4(1):72–4.

12. Wang J, Xian H, Licis A, Deych E, Ding J, McLeland J, et al. Measuring the impact of apnea and obesity on circadian activity patterns using functional linear modeling of actigraphy data. J Circadian Rhythms [Internet]. 2011 Oct 13 [cited 2025 May 30];9:11. Available from: https://pmc.ncbi.nlm.nih.gov/articles/PMC3245508/

13. Crainiceanu CM, Goldsmith J, Leroux A, Cui E. Functional Data Analysis with R [Internet]. First. Chapman and Hall/CRC, editor. 2023. Available from: https://www.crcpress.com/Chapman--HallCRC-Monographs-

14. Filardi M, Gnoni V, Tamburrino L, Nigro S, Urso D, Vilella D, et al. Sleep and circadian rhythm disruptions in behavioral variant frontotemporal dementia. Alzheimer’s and Dementia. 2024 Mar 1;20(3):1966–77.

15. Miyauchi K, Fujimoto K, Abe T, Takei M, Ogawa K. Cross-sectional assessment of sleep and fatigue in middle-aged Japanese women with primary Sjogren syndrome or rheumatoid arthritis using self-reports and wrist actigraphy. Medicine [Internet]. 2021 Sep 17 [cited 2026 Jan 9];100(37). Available from: https://pubmed.ncbi.nlm.nih.gov/34664865/
